# Macular choroidal thickness in normal Egyptians measured by swept source optical coherence tomography

**DOI:** 10.1186/s12886-016-0314-1

**Published:** 2016-08-05

**Authors:** Magdy Moussa, Dalia Sabry, Wael Soliman

**Affiliations:** 1 Ophthalmology Department, Tanta University, Tanta, Egypt; 2 Mansoura Ophthalmic Center, Mansoura, Egypt; 3 Ophthalmology Department, Assiut University Hospitals, Assiut, Egypt

**Keywords:** Choroidal thickness, Deep-range imaging swept source, OCT, Egypt

## Abstract

**Background:**

To provide a normal database of choroidal thickness (CT) in nine Early Treatment Diabetes Retinopathy Study (ETDRS) subfields in Egypt using deep-range imaging swept source optical coherence tomography (DRI SS OCT).

**Methods:**

This study included a total of 129 eyes of 71 normal Egyptian subjects, comprising 63 males and 66 females. The mean age was 36.85 ± 14.22 years (range, 16–67 years). The mean axial length was 23.84 ± 0.78 mm. CT was measured in nine subfields as defined by the ETDRS-style grid using a DRI SS OCT, and line measurements of subfoveal choroidal thicknesses (SFCT) were also performed.

**Results:**

Mean SFCT was 300.87 ± 72.256 μm for ring measurements and 319.72 ± 76.45 μm for line measurements (*P* = 0.04). CT was higher in the superior and temporal quadrants than the inferior and nasal quadrants. A negative correlation between subfoveal choroidal thickness and age was detected in all regions (*P* < 0.001) except the nasal quadrant. A negative correlation between the SFCT and axial length was also detected (*P* < 0.001). Males tended to have a thicker choroid than females; however, the difference was not significant.

**Conclusions:**

DRI SS OCT provides a topographic map of choroidal thickness with an ETDRS layout. This study establishes, for the first time, a normal database for CT in the Egyptian population. Age and axial length were associated with choroidal parameters in healthy subjects. Line measurements of the SFCT differed significantly from SFCT ring measurements, so it is recommended that each method be compared independently.

**Electronic supplementary material:**

The online version of this article (doi:10.1186/s12886-016-0314-1) contains supplementary material, which is available to authorized users.

## Background

The choroid plays a central role in ocular metabolism, temperature control, and volume regulation [1, 2]. Choroidal thickness has been reported to change with smoking, arterial pressure, daytime, axial length, and age [3–7]. However, the choroid is also involved in the pathogenesis of several vision-threatening disorders, such as age-related macular degeneration (AMD) [8], central serous chorioretinopathy [9], polypoidal choroidal vasculopathy [10], the Vogt–Koyanagi–Harada disease [11], and high myopia-related chorioretinopathy [12]. These disorders show the need for understanding the choroidal structure in ocular diseases and the importance of having a normative database of choroidal thicknesses (CT).

Accurate morphological evaluation of the choroid using spectral-domain OCT (SD-OCT) is not possible because of its posterior location and the scattering of light caused by pigmented retinal pigment epithelium (RPE) cells. Spaide et al. developed an enhanced depth imaging (EDI) OCT, which enables in vivo cross-sectional imaging of the choroid and measurement of the thickness of the choroid [13]. OCT with longer wavelength light sources has recently been developed [14, 15], which allows better penetration and improved choroidal visualization. Furthermore, swept source OCT (SS-OCT) with a 1050-nm wavelength has been used to study the choroid in normal [3, 16] and diseased eyes [12, 17, 18], resulting in more detailed images [19, 20].

The present study was designed to study CT in the normal Egyptian population, using deep-range imaging swept source optical coherence tomography (DRI SS OCT). The study determined the baseline CT in nine macular Early Treatment Diabetes Retinopathy Study (ETDRS) subfields, and evaluated the relationship of the CT with age, axial length, and sex. In addition, the study compared the line measurements of subfoveal choroidal thicknesses (SFCT) with those automatically calculated in the central 1-mm ring in the ETRDRS-style grid.

## Methods

This prospective study was performed from December 2014 to March 2015, with normal Egyptian volunteers 16–67 years of age. The subjects were recruited from the outpatient clinic of the Ophthalmology Department, Tanta University, Tanta, Egypt. All subjects referred to themselves as healthy. The study was conducted in accordance with the Declaration of Helsinki and its subsequent amendments. Explanation of the nature of the study was given to the subjects. Informed written consent was obtained from all individual participants in the study. The study approved by ethical committee of Tanta faculty of medicine.

Exclusion criteria were: 1) Myopia or hyperopia more than 3 diopters (D), 2) eyes with dystrophic or degenerative diseases, 3) prior ocular surgery (with the exception of uncomplicated cataract surgery) or laser therapy, 4) anterior or posterior segment inflammation, 5) glaucoma, 6) eyes with choroidal abnormalities or conditions that could affect their thickness such as central serous chorioretinopathy, nevus, pregnancy, malignant hypertension, or haemangiomas, 7) patients with diabetes, non malignant hypertension and smoking and 8) patients with optical media opacity that significantly disturbed OCT image acquisition.

A complete ophthalmic examination was performed on all patients that included measurement of best-corrected visual acuity (BCVA), intraocular pressure (IOP) measurement using a Goldmann applanation tonometer, anterior segment examination with a slit lamp, dilated fundus examination, refraction using an Autorefractor KR-8900 (Topcon, Tokyo, Japan), and axial length measurement using an IOL Master (Carl Zeiss Meditec, Dublin, CA, USA).

Choroidal thickness measurements were performed using a Topcon DRI-1 Swept Source SSOCT (Topcon, Tokyo, Japan). It is the first swept source OCT developed for posterior segment imaging of the eye. A 1050-nm wavelength light source, a scanning speed of 100,000 A scans/second [twice that of spectral domain (SD) OCT], and a 5 μm resolution were used. All these properties facilitated uniform scanning sensitivity and superior visualization of the vitreous and choroid in the same scan.

After pupillary dilation with 1 % tropicamide and 2.5 % phenylephrine hydrochloride, a 12, 9-mm radial line scan protocol was applied. Each radial line was automatically scanned repeatedly, 32 times in the same position, and then 12 high-resolution averaging B-scan images were produced. Each scan was reviewed to be sure it is centered on the fovea. Only good-quality scans were included (five eyes were excluded from the study because of poor image quality).

**Fig. 1:**
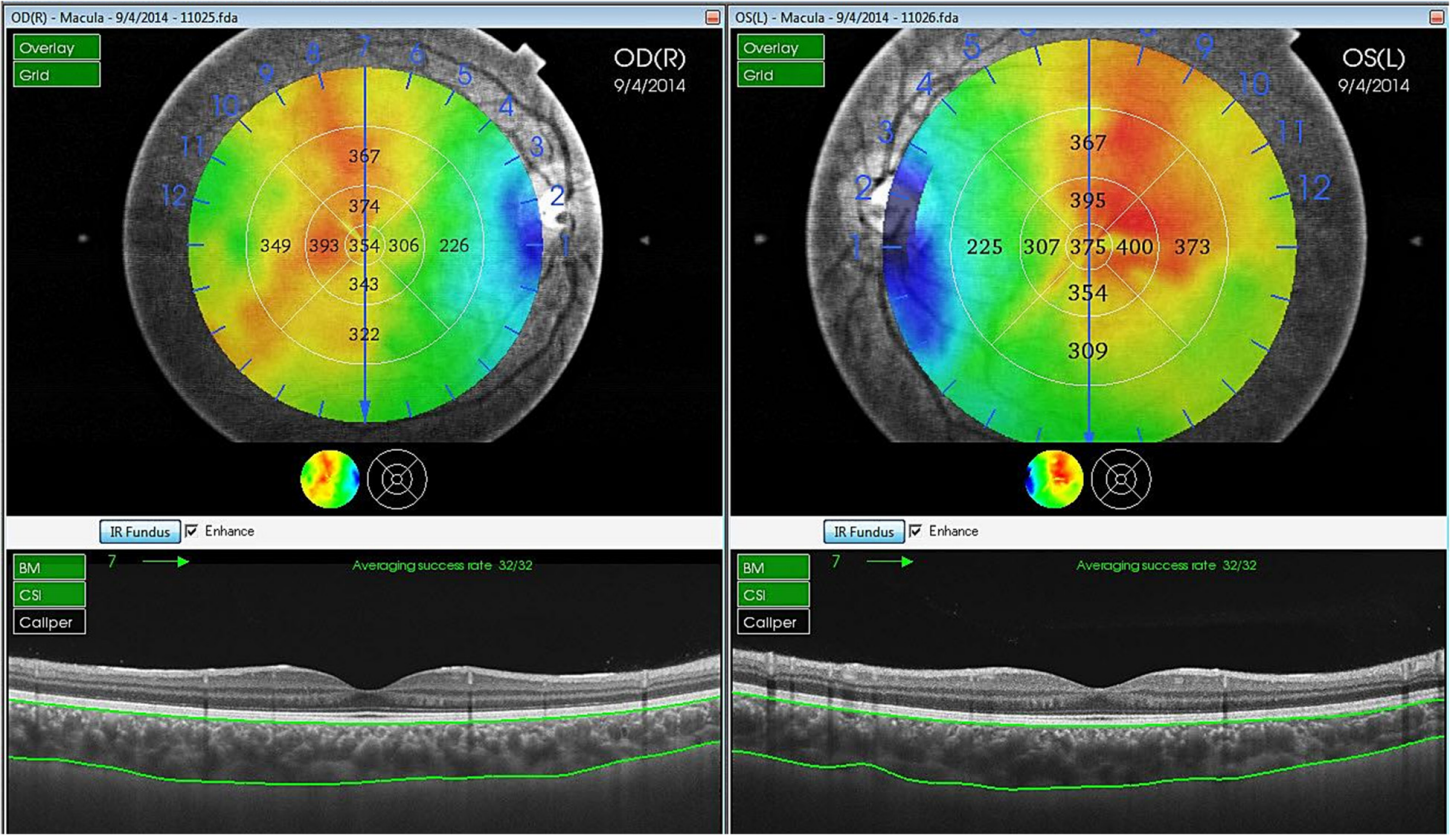
Automatically plotted reference lines and the colored topographic map of the nine subfields as defined by the Early Treatment Diabetes Retinopathy Study-style grid in both eyes

**Fig. 2:**
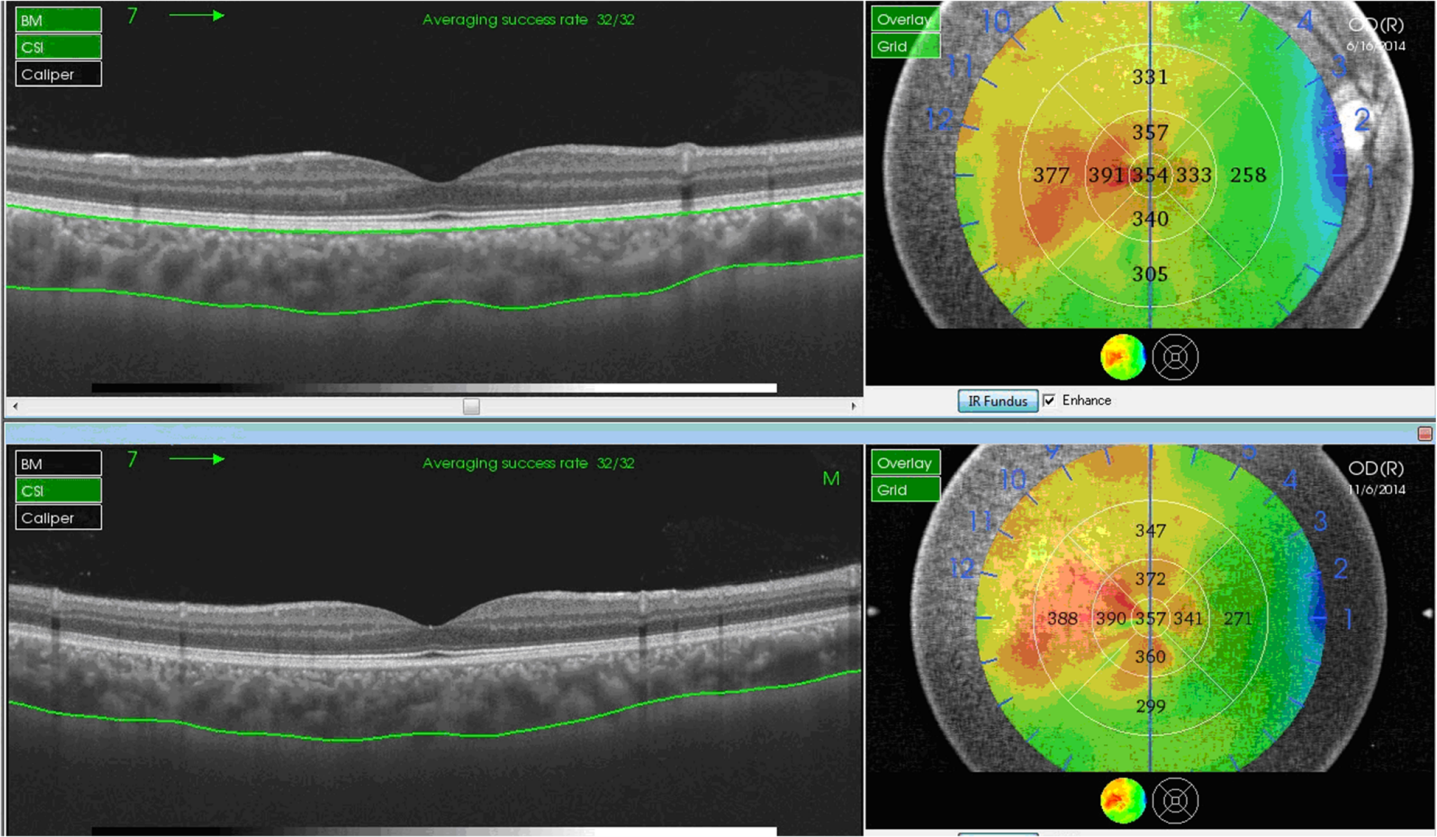
Change in choroidal thickness before and after manual line adjustment

Choroidal thickness was defined as the perpendicular distance between the posterior edge of the hyper-reflective RPE and the choroid/sclera junction. Using the built-in mapping software, choroidal thickness was automatically calculated and shown as a colored topographic map with nine subfields defined by the ETDRS-style grid. A three-dimensional topographic map of thicknesses was then generated. The 9 automatically calculated ETDRS subfields comprised: the SFCT in the inner ring, nasal inner macula, superior inner macula, temporal inner macula, inferior inner macula, nasal outer macula, superior outer macula, temporal outer macula, and inferior outer macula, which were surrounded by rings 1, 3, and 6 mm in diameter (Fig. 1). For segmentation of the choroidal boundaries, the built-in segmentation modifying tool was used by a well-trained analyst (M.M.). The reference lines of the retinal boundary [retinal pigment epithelium (RPE)] and the choroidal-scleral boundary were automatically plotted in each set of 12 radial scans. Then each line was inspected individually for any misalignment and corrected manually, if necessary. Figure 2 shows the change in choroidal thickness before and after manual line adjustment.

**Fig. 3:**
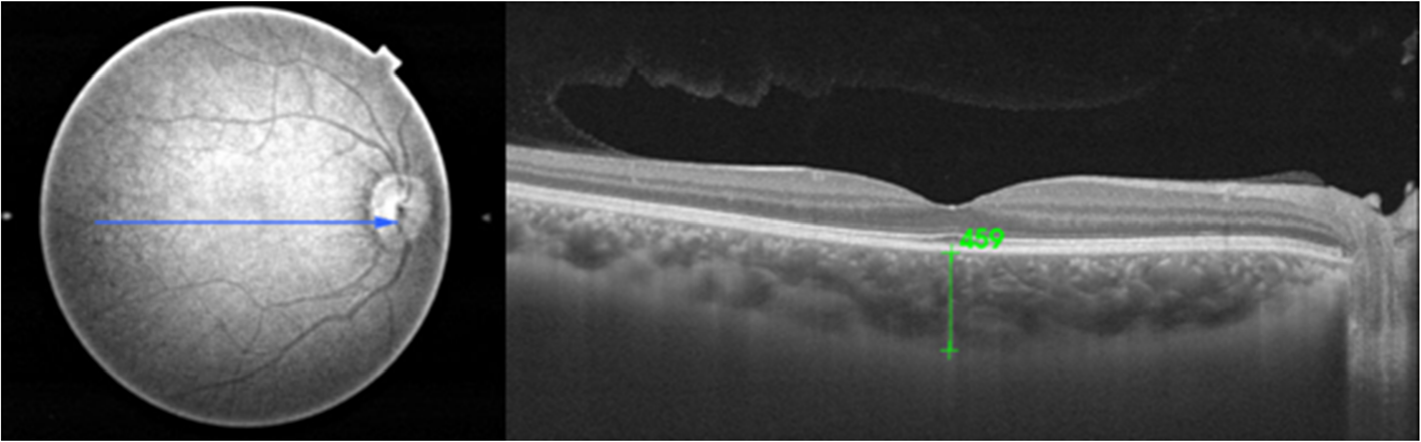
Line measurement of the central subfield choroidal thickness

Line measurements of the SFCT were performed manually by calculating the average measurements of vertical and horizontal line scans passing through the foveal center. The measurements were performed manually from the posterior edge of the hyper-reflective RPE to the choroid/sclera junction (Fig. 3).

Three-dimensional (3D) optic disc scanning (optic disc protocol) and a 3D macular scanning (macula protocol) were performed at the same session. The optic disc protocol was performed to exclude any peripapillary nerve fiber layer thinning or disc abnormality, and the macular protocol was performed to exclude any macular abnormalities.

All measurements were performed at the same time of day.

### Statistical analyses

Statistical analyses were performed using the Statistical Package for the Social Sciences (SPSS 20.0; SPSS, Chicago, IL, USA). The Kolmogorov-Smirnov test was used to assess the normal distribution of the results, and the paired *t-*test was used to compare means between areas. To calculate the correlation among the study values, Pearson’s correlation coefficients were calculated. Choroidal thicknesses were compared between axial lengths, sex, and age using the Student’s *t-*test. Choroidal values for the nine areas defined in the ETDRS were compared to assess the differences in the various areas. Values of *P* ≤ 0.05 were considered statistically significant.

## Results

This prospective study included a total of 129 eyes of 71 normal Egyptian subjects. Thirteen eyes were excluded from the study (eight because of refractive errors belonging to the excluded range and five because of significant media opacity). Patients comprised 63 (48.8 %) males and 66 (51.2 %) females. The mean age was 36.85 ± 14.22 (range, 16–67 years). The mean axial length was 23.84 ± 0.78 mm (range, 22.7–25.6 mm) and the mean spherical equivalent was -0.9D (range, -2.5– + 1.25 D). The mean IOP was 18.7 (range, 14.8–19.5 mmHg). All subjects had a BCVA of ≥0.8 and all subjects were Egyptians.

The mean CT in the different subfields is listed in Table 1. Mean SFCT was 300.87 ± 72.26 μm for the ring measurements, and 319.72 ± 76.45 μm for the line measurements. The CT was greater in the superior and inferior subfields (3-mm ring) compared with the temporal and nasal subfields of the same ring, and it was greater in the superior and temporal subfields (6-mm ring) compared with the inferior and nasal subfields of the same ring.

**Table 1:** Mean choroidal thickness in healthy Egyptians evaluated by the Early Treatment Diabetic Retinopathy Study grid

Areas	Choroidal thickness (μm)		
	Mean ± SD	Minimum	Maximum
Central subfoveal ring	300.87 ± 72.256	157	470
Central subfoveal line	319.72 ± 76.45	152	530
Inferior inner macula	297.67 ± 77.08	129	480
Superior inner macula	306.76 ± 75.05	167	487
Nasal inner macula	282.74 ± 71.33	109	455
Temporal inner macula	293.47 ± 73.86	109	472
Inferior outer macula	279.19 ± 77.60	117	481
Superior outer macula	298.13 ± 73.89	172	467
Nasal outer macula	239.39 ± 73.83	79	430
Temporal outer macula	288.44 ± 73.07	117	475

*SD* standard deviation

**Fig. 4:**
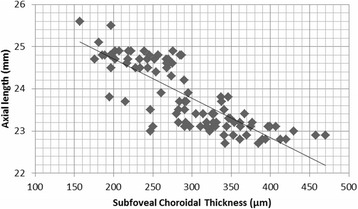
Scatter plot of the axial length and subfoveal choroidal thickness

Table 2 shows the differences among the nine subfields. A statistically significant difference (*P* < 0.05) was found between the temporal and nasal subfields (3 and 6-mm regions) and between the center and nasal 3-mm subfields. The best correlation was found between the temporal and nasal 3-mm subfields (*P <* 0.001). In addition, a negative correlation was found between the SFCT and axial length (*r* = -0.76, *P* < 0.001). The CT tended to decrease with an increase in axial length (Fig. 4).

**Table 2:** Differences between mean thicknesses of the areas evaluated and statistical comparison of the results

Area	Choroidal thickness (μm)	
	Mean ± SD	*P*
Temporal (6 mm)/nasal (6 mm)	48.33 ± 9.2	<0.001
Superior (6 mm)/inferior (6 mm)	18.95 ± 9.435	0.05
Temporal (3 mm)/nasal (3 mm)	21.44 ± 9.12	0.02
Superior (3 mm)/inferior (3 mm)	9.10 ± 9.47	0.34
Temporal (3 mm)/center (1 mm)	20.29 ± 18.87	0.28
Nasal (3 mm)/center (1 mm)	-18.12 ± 8.94	0.04
Superior (3 mm)/center (1 mm)	5.89 ± 9.17	0.75
Inferior (3 mm)/center (1 mm)	-3.20 ± 9.30	0.73

*SD* standard deviation

Differences between the males (*n* = 63) and females (*n* = 66) were also evaluated. Males had a thicker choroid than females in all subfields; however, there was no statistically significant difference except in the inferior outer subfields (*P* = 0.05) (Table 3). A negative correlation between subfoveal choroidal thickness and age was detected in all subfields (*P <* 0.001) except the nasal inner and outer subfields (Table 4). Line measurements of the SFCT showed a significant difference from the ring measurements (difference, 18.85 ± 7.46 μm, *P* = 0.04).

**Table 3:** Mean choroidal thickness of male and female normal Egyptians

Area	Choroidal thickness (μm)		
	Male Mean ± SD	Female Mean ± SD	*P*
Central ring	309.22 ± 62.488	292.89 ± 80.152	0.20
Inferior inner region	308.68 ± 67.849	287.15 ± 84.144	0.11
Superior inner region	316.89 ± 62.394	297.11 ± 84.76	0.14
Nasal inner region	289.84 ± 62.081	275.97 ± 79.038	0.27
Temporal inner region	311.49 ± 61.712	297.21 ± 85.791	0.28
Inferior outer region	293.11 ± 68.341	265.89 ± 83.887	0.05
Superior outer region	310.06 ± 66.709	286.74 ± 78.987	0.07
Nasal outer region	246.41 ± 66.633	232.68 ± 80.031	0.29
Temporal outer region	296.98 ± 61.591	280.29 ± 82.211	0.19

Male axial length was 23.88 ± 0.49 mm, and female axial length was 24.12 ± 0.21 mm (*P* = 0.11)

*SD* standard deviation

**Table 4:** Variation with age of the mean choroidal thickness

Area	Choroidal thickness (μm)		
	<40 years Mean ± SD	≥40 years Mean ± SD	*P*
Central ring	320.32 ± 73.93	276.30 ± 62.48	<0.0001
Inferior inner region	319.53 ± 77.31	270.05 ± 67.92	<0.0001
Superior inner region	327.24 ± 78.16	280.91 ± 62.54	<0.0001
Nasal inner region	296.44 ± 72.74	265.44 ± 66.15	0.01
Temporal inner region	329.15 ± 76.69	272.65 ± 60.04	<0.0001
Inferior outer region	303.81 ± 77.02	248.09 ± 66.94	<0.0001
Superior outer region	320.85 ± 77.72	269.44 ± 57.63	<0.0001
Nasal outer region	250.51 ± 71.98	225.33 ± 74.36	0.05
Temporal outer region	313.68 ± 75.09	256.56 ± 56.59	<0.0001

*SD* standard deviation

## Discussion

The SS-OCT uses a longer wavelength source than the SD-OCT, which facilitates accurate visualization of the chorioscleral interface in normal eyes. Thus, choroidal thickness can be measured accurately.

In the present study, the macular subfields of healthy Egyptian volunteers were viewed with a DRI SS OCT using a wavelength of 1050 nm. A 12 radial scan protocol enabled a reliable topographic map of choroidal thickness with an ETDRS layout. This protocol automatically produced high-resolution, averaged B-scan images, which were clear enough to visualize the choroidal boundary and as accurate as the single-line scan protocol. The protocol required semi-automated adjustment of the retinal-choroidal boundary in each of the 12 radial B-scans that generated a topographic map of the choroidal thickness. All procedures were performed using built-in software available with the DRI SS OCT. The choroid and anatomical landmarks of the two reference lines were clearly visible in all the study cases.

Numerous studies have measured the CT of normal subjects. Most of these studies measured the mean SFCT, and some reported values of less than 300 μm [5, 16, 20–22], while others reported values greater than 300 μm [3, 13, 23–26]. Our mean SFCT was 300.87 ± 72.26 μm for ring measurements while it was greater (319.72 ± 76.45 μm) for line measurements (*P* = 0.04). This could be explained by the difference in the nature of the measurements. The line protocol measures the thickest central point while the ring measures the entire 1-mm ring. The findings showed that the two methods differed, with higher values obtained by the line method. It is therefore better to compare the SFCT values obtained by each method. Our mean age was 36.85 ± 14.22 years. Ikuno et al. [16] reported an approximate SFCT of 354 μm in 43 Japanese subjects with a mean age of 39.4 years using a 1060 nm-based light source. Sanchez-Cano et al. [27] reported a mean SFCT of 345.67 μm in normal Caucasian subjects with a mean age of 24 years. However, Ding et al. [21] measured the CT thickness in normal Chinese subjects and reported a mean SFCT of 294.63 ± 75.90 μm in subjects less than 60 years of age. Taken together, these findings suggest that SFCT varies with both age and race.

In our study, we found that the choroid is thickest in the superior and temporal subfields. This finding is consistent with numerous previous reports [4, 5]. In our study, the temporal choroid was significantly thicker than the nasal choroid in both the 3 and 6-mm rings (*P =* 0.02 and *P* <0.001, respectively). Many previous studies also reported that the CT decreases in the nasal quadrant and that the temporal choroid is significantly thicker than the nasal quadrant [5, 20–24]. A significant difference in thicknesses was also detected when comparing the superior and inferior 6-mm subfields (*P =* 0.05) and 3-mm nasal and central subfields (*P* = 0.04).

Overall, no correlation was found between CT and sex. Males tended to have a thicker choroid than females in all subfields; however, no statistically significant difference was found except in the inferior outer subfield (*P* = 0.05). These findings are consistent with those reported by Park et al. [24], who reported no significant difference between the sexes and SFCT. However, Manjunath et al. [22] reported a slightly greater thickness in males than females.

In our study there was a significant negative correlation between SFCT and axial length (*P* < 0.001), which has also been reported in previous studies [3–5]. We identified a -0.76 μm/mm factor that is greater than reported by previous studies [5, 16, 25, 28].

We found a strong negative correlation between age and CT. A significant difference was found in all ETDRS subfields between younger subjects and subjects older than 40 years of age. The least significant difference was detected in the nasal subfield, because it is already the thinnest area of the choroid. Other studies have also reported a reduction in CT with age [16, 29]. The thinning of the choroid with age reduces its ability to supply oxygen and other metabolites to the RPE and outer retina, which may partially explain the occurrence of age-related macular degeneration.

## Conclusion

Our study provides a database for the CT in the normal Egyptian population. DRI SS OCT provides a topographic map of choroidal thickness with an ETDRS layout. The resulting images can visualize the choroidal boundary, which sometimes requires semi-automated adjustment of the retinal-choroidal boundary. Age and axial length were associated with choroidal parameters in normal subjects, and line measurements of the SFCT differed significantly from the SFCT ring measurements, so it is recommended to compare each method independently.

## Abbreviations

CT, Choroidal thickness; DRI SS OCT, Deep-range imaging swept source optical coherence tomography; ETDRS, Early Treatment Diabetes Retinopathy; SD-OCT, Spectral-domain OCT; SFCT, Subfoveal choroidal thicknesses

## Additional file

Additional file 1: Choroidal thickness, retinal thickness, choroidal thickness 1. (XLSX 40 kb)
